# Improving healthcare value: Optimizing billing and documentation for inpatient addiction consultation services

**DOI:** 10.1002/jhm.70461

**Published:** 2026-09-06

**Authors:** Philip A. Durney, Nancy McLaughlin, Susan L. Calcaterra

**Affiliations:** ^1^ Department of Medicine, Division of Hospital Medicine Thomas Jefferson University Philadelphia Pennsylvania USA; ^2^ Clinical Documentation Improvement Program Thomas Jefferson University Philadelphia Pennsylvania USA; ^3^ Department of Medicine, Division of Hospital Medicine University of Colorado Aurora Colorado USA; ^4^ Department of Medicine, Division of General Internal Medicine University of Colorado Aurora Colorado USA

## Abstract

Patients with substance use disorder (SUD) represent a high‐complexity, high‐cost population on hospital medicine services, yet their admissions are systematically under‐reimbursed due to narrow clinical documentation. Increasingly, hospital‐based addiction consultation service (ACS) clinicians provide dedicated addiction care to medically complex hospitalized patients. Hospitalizations coded solely for SUD fail to capture clinical complexity and concomitant diagnoses, depressing diagnosis‐related group weights and Case Mix Index. We describe three actions for ACS clinicians, hospitalists, and system leaders: (1) document the complete clinical picture, (2) utilize advanced billing mechanisms, and (3) build institutional infrastructure to improve reimbursement to support long‐term ACS sustainability.

## CLINICAL SCENARIO

A 50‐year‐old man is admitted with encephalopathy, fever, cough, and respiratory distress. He drinks 24 beers daily, with his last drink 18 h prior. He is febrile (100.3 °F), hypertensive, tachycardic, and his oxygen saturation is 90% on room air. He has tongue fasciculations, tremors, and is oriented only to self. Laboratory results show a leukocytosis of 15,000, sodium of 130, lactate of 1.4, a blood alcohol level of 99, and a creatinine of 2.4 (baseline 0.8) despite fluid resuscitation. Chest X‐ray reveals a right lower lobe infiltrate. The hospitalist consults the addiction consultation service (ACS) to manage alcohol withdrawal and alcohol use disorder (AUD).

The ACS clinician documents: “AUD with withdrawal. Recommend CIWA protocol and naltrexone initiation.” The hospitalization is coded as all patient refined (APR)‐diagnosis‐related group (DRG) 775, severity of illness level (SOI) 1 (minor), and reimbursement of $4029. Had the clinician also documented “aspiration pneumonia due to alcohol withdrawal, metabolic encephalopathy, acute hypoxic respiratory failure, and acute tubular necrosis,” the hospitalization would shift to APR‐DRG 775 SOI level 4 (extreme), with a reimbursement of $25,898, a difference of $20,000 for the same clinical care (Table 1). This example illustrates the importance of complete documentation, as coders may use documented diagnoses by any treating clinician to inform reimbursement. What should ACS clinicians and hospitalists document to ensure appropriate reimbursement?

**Table 1:** Difference in DRG impact based on clinician documentation for two hospitalized patients with substance use disorder (SUD) and medical comorbidities.

DRG	Description	Example comorbidities	Relative weight	Length of stay	Estimated reimbursement^a^
**Patient 1. Hospitalized patient with alcohol use disorder (AUD) and alcohol withdrawal with narrow versus optimized documentation and reimbursement**					
MS‐DRG^b^ 897 (Narrow)	AUD w/o MCC, withdrawal	Only primary AUD documented	0.8828	3.3	$9,864
MS‐DRG^c^ 896 (Optimized)	AUD with MCC	Aspiration pneumonia, metabolic encephalopathy, acute hypoxic respiratory failure, acute tubular necrosis, hyponatremia, and AUD	1.7451	4.8	$18,869
APR‐DRG 775 SOI^d^ 1 (Narrow)	AUD, minimal secondary dx	Only primary AUD documented	0.4501	2.5	$4,029
APR‐DRG 775 SOI 3	AUD, full comorbidity capture	Aspiration pneumonia, metabolic encephalopathy, acute hypoxic respiratory failure, hyponatremia, and AUD	1.2404	4.1	$11,104
APR‐DRG 775 SOI 4 (Optimized)	AUD, full comorbidity capture	Aspiration pneumonia, metabolic encephalopathy, acute hypoxic respiratory failure, hyponatremia, acute tubular necrosis, and AUD	2.8929	9.6	$25,898
**Patient 2. Hospitalized patient with opioid use disorder (OUD) with narrow versus optimized documentation and reimbursement**					
MS‐DRG 897 (Narrow)	OUD w/o MCC, withdrawal only	Only primary OUD documented	0.8828	3.3	$9,864
MS‐DRG 896 (Optimized)	OUD with MCC	SIRS, acute kidney injury, metabolic acidosis, homelessness, and OUD	1.7451	4.8	$18,869
APR‐DRG 773 SOI 1 (Narrow)	OUD, minimal secondary dx	Only primary OUD documented	0.3534	3.1	$3,163
APR‐DRG 773 SOI 3 (Optimized)	OUD, full comorbidity capture	SIRS with acute organ dysfunction, acute kidney injury, metabolic acidosis, unsheltered homelessness, and OUD	0.9822	3.9	$8,793

Abbreviations: AUD, alcohol use disorder; APR‐DRG, All Patient Refined‐Diagnosis‐Related Group; MS‐DRG, Medicare Severity–Diagnosis‐Related Group; OUD, opioid use disorder; SIRS, Systemic Inflammatory Response Syndrome; SOI, severity of illness.

^a^ Estimated payment = base rate x relative weight, using an illustrative base rate of $8,952 for the APR‐DRG rows; MS‐DRG estimates reflect Medicare payment methodology. DRG assignment, severity of illness, relative weights, length of stay, and estimated reimbursement were calculated using proprietary reimbursement calculation software. APR‐DRG reimbursement methodologies are established by individual state Medicaid agencies and certain commercial payers; APR‐DRG values are based on Pennsylvania Medicaid APR‐DRG v43 (FY26). Base rates vary widely across health systems, and actual payment varies by hospital specific base rates, wage index adjustment, payer, and outlier provisions.

^b^ MS‐DRG rows show geometric mean length of stay.

^c^ APR‐DRG rows show average length of stay.

^d^ Complication or comorbidity (CC) and major complication or comorbidity (MCC) designations apply only to the MS‐DRG system; the APR‐DRG system instead assigns a severity of illness level from 1 (minor) to 4 (extreme).

## INTRODUCTION

Hospitalizations involving substance use disorders (SUDs) are among the most common and costly on hospital medicine services, exceeding $13 billion annually in the United States (US).^1^ Hospitalists serve as the attending of record for most SUD admissions, and these patients typically have higher readmission rates, longer stays, and more serious medical complications than other hospitalized patients.^2^, ^3^ ACS programs reduce readmissions, improve abstinence, and lower mortality, yet rely primarily on evaluation and management (E/M) billing, or service‐based charges tied to clinician time and clinical complexity, often requiring institutional subsidies or grants to remain financially sustainable.^4^, ^5^, ^6^

Hospital care for patients with SUD generates two distinct payment streams. *Facility reimbursement* compensates the hospital. Coders translate clinician documentation into International Classification of Disease Codes (ICD)−10‐Clinical Modification (CM) codes that group the hospitalization into a DRG. The DRG relative weight multiplied by the hospital's base rate determines facility payment and contributes to the case mix index (CMI). *Professional reimbursement* compensates physicians and advanced practice clinicians (APCs) based on their own documentation. Accurate documentation strengthens both payment streams, and for hospitals with an ACS, it helps demonstrate the clinical complexity and financial value that support ACS sustainability.

Coding relies entirely on clinician documentation (“clinician” throughout refers to any independently billing physician or APC). Hospital coders assign ICD‐10 codes based on what is documented and cannot infer diagnoses. A clinical documentation integrity (CDI) specialist may only query the clinician about undocumented conditions before the chart closes; they cannot create documentation. Because coders may use diagnoses documented by any physician or APC involved in a patient's care, ACS documentation strengthens the shared record by capturing comorbidities other clinicians may not recognize or document.^7^ A grouping algorithm assigns each hospitalization, that is, the full inpatient episode of care, a single DRG. Two systems are commonly used: Medicare Severity (MS)‐DRGs, which stratify by the presence of complications or comorbidities (CC/MCC), and APR‐DRGs, which assign a severity of illness score (SOI) from 1 (minor) to 4 (extreme).

SUD, particularly opioid use disorder (OUD), are routinely underdiagnosed and under documented in hospital records.^8^, ^9^ ICD‐10 comorbidity coding is rarely taught in medical training, and CDI specialists may lack familiarity with SUD‐specific comorbidity patterns. This results in documentation gaps following chart closure resulting in “narrow” documentation, lower DRGs, and depressed CMI, underrepresenting the complexity of care that ACS clinicians and hospitalists provide, thus weakening the financial argument for ACS program sustainment. As shown in Table 1, optimized documentation for AUD‐ and OUD‐related hospitalizations can increase reimbursement by $3,000–$25,000 without changing the care delivered.

Accurate documentation impacts three groups: *patients*, by sustaining access to hospital‐based addiction care that may represent an opportunity for recovery; *clinicians*, by supporting reimbursement that reflects cognitive intensity; and *hospitals*, by ensuring DRG assignment captures the complexity and value of care provided to hospitalized patients with SUD, highlighting ACS programs as value‐generating. Documentation incentives vary by setting. Hospitals benefit from accurate DRG‐based facility payments, while relative value unit (RVU)‐based clinicians benefit through professional billing and work RVU productivity capture. Independent community physicians receive revenue mainly through professional fees and may not share in hospital‐level payment gains. Thus, documentation improvement creates both direct clinician and institutional incentives that health systems should align.

## SUMMARY OF ACTIONS TO IMPROVE HEALTHCARE VALUE

The following actions target three audiences: Actions 1 and 2 target ACS clinicians and hospitalists; Action 3 targets leaders responsible for documentation infrastructure, including ACS and hospital medicine leaders, CDI specialists, and revenue cycle teams. Each draws on evidence from inpatient documentation improvement initiatives.

### Action 1: Document a comprehensive clinical picture tied to SUD (facility reimbursement: DRG and CMI)

Documenting every diagnosis identified, evaluated, and addressed, including the SUD, is among the most impactful ways to optimize reimbursement. SUD‐related hospitalizations often involve infectious, end‐organ, and metabolic comorbidities that carry CC/MCC status and affect DRG weight. Documentation should name the diagnosis, provide supporting objective data, that is, laboratory values, vital signs, imaging, and a description of how it was addressed. The note should also capture problems addressed, clinical reasoning, and risk to support high‐complexity E/M billing. Note templates with SUD‐specific comorbidity prompts have increased MCC capture rates by up to 16% among patients receiving care from surgeons and hospitalists.^10^, ^11^, ^12^

### Action 2: Apply advanced billing codes when appropriate (professional fee reimbursement)

Beyond standard E/M billing, clinicians often underuse critical care and prolonged services billing. Critical care billing (CPT 99291‐99292) applies to ≥30 min managing life‐threatening organ dysfunction based on clinical conditions and treatment intensity, regardless of the treatment unit (acute care vs. critical care). Severe alcohol withdrawal with delirium tremens, autonomic instability, and hemodynamic compromise from drug toxicity all qualify as critical care billing independent of hospital location. Additionally, multiple specialists may bill critical care on the same day when managing distinct diagnoses.^13^ Prolonged services billing (CPT 99418; HCPCS G0316 for Medicare) apply when clinician time substantially exceeds a standard visit.^14^ Structured education within a pediatric hospitalist service increased appropriate critical care codes billing from 21% to 74%, quadrupling estimated reimbursement.^15^ Both billing mechanisms require knowledge of accurate documentation of eligibility and time spent in care delivery.

### Action 3: Build an infrastructure that makes improvement sustainable (both payment streams)

Clinician education alone rarely produces durable change without institutional support. CDI specialists can identify missing or unclear diagnoses, but effective review of SUD‐related hospitalizations requires familiarity with SUD‐specific conditions and is rarely part of CDI training. Aligning documentation between ACS consultants and the primary service is crucial for record accuracy. CDI specialists query clinicians to clarify ambiguous, inconsistent, or conflicting documentation. Front‐end strategies, including CDI education on SUD‐related comorbidities and shared querying protocols, reduce administrative burden and coding delays while reinforcing documentation habits among hospitalists and ACS clinicians systemwide. Revenue cycle dashboards tracking DRG assignment and CMI from hospitalizations with ACS involvement make the financial impact visible to leadership, setting the stage for resource investment. A 2016 national survey found that 90% of hospitals with structured CDI programs saw reimbursement increase by $1.5 million through improved CMI capture.^16^ Periodic audits distinguish accurate documentation from upcoding, a fraudulent billing practice where clinicians submit codes for more expensive, complex, or severe diagnoses than were documented.

## IMPLICATIONS FOR SUSTAINABILITY AND DISSEMINATION

These actions are scalable across institutions, regardless of ACS presence. Professional societies, including the Society of Hospital Medicine, could develop standardized ACS billing curricula and shared CDI protocols for SUD‐related comorbidities. Multisite studies are needed to determine whether improved documentation translates into higher DRG assignment and greater ACS growth and impact.

## BALANCING MEASURES

The primary risk of a documentation improvement initiative is inadvertent upcoding, or coding diagnoses that are not clinically supported. Each coded diagnosis must be supported by objective data and clinician documentation. Note templates and CDI support mitigate documentation burden, but durable change requires recurring education rather than one‐time training. Time‐based billing and prolonged services billing applies only to clinicians who bill independently; contributions from social workers, pharmacists, and nurses are captured through program‐level value metrics.

## CONCLUSIONS AND NEXT STEPS

The differences between the notes in the clinical scenario are descriptive, not clinical; one captures the patient's full medical complexity, the other does not. Incomplete documentation can cost hospitals thousands per hospitalization and weaken the financial foundation of ACS programs. For hospitalists, these documentation principles are immediately actionable and relevant to program financial health. For patients, a financially viable ACS means continued access to SUD treatment. For hospitals, accurate documentation corrects systematic underrepresentation of SUD complexity in CMI and quality metrics. The roadmap is clear: standardize billing curricula, invest in CDI infrastructure, and build evidence linking documentation to ACS program survival. Hospitalists and ACS clinicians who master these actions champion clinical excellence and financial sustainability, advancing ACS as indispensable inpatient care.

## CONFLICT OF INTEREST STATEMENT

The authors declare no conflicts of interest.

## Data Availability

Data sharing is not applicable to this article as no datasets were generated or analysed during the current study.
